# Ultrasound-based nomogram for postpartum hemorrhage prediction in pernicious placenta previa

**DOI:** 10.3389/fphys.2022.982080

**Published:** 2022-08-22

**Authors:** Yangzi Zhou, Zixuan Song, Xiaoxue Wang, Mingjie Zhang, Xueting Chen, Dandan Zhang

**Affiliations:** ^1^ Department of Obstetrics and Gynecology, Shengjing Hospital of China Medical University, Shenyang, China; ^2^ Department of Health Management, Shengjing Hospital of China Medical University, Shenyang, China; ^3^ Department of Surgery, Shengjing Hospital of China Medical University, Shenyang, China

**Keywords:** pernicious placenta previa, postpartum hemorrhage, nomogram, univariate logistic regression, LASSO regression, decision curve analysis, area under the curve

## Abstract

**Background:** Pernicious placenta previa (PPP) is one of the most dangerous complications in pregnancy after cesarean section, with high perinatal mortality. This study aimed to develop a nomogram to predict postpartum hemorrhage in patients with PPP.

**Methods:** A total of 246 patients with confirmed PPP at Shengjing Hospital of China Medical University from January 2018 to December 2021 were included. Patients were divided into to two cohorts depending on a postpartum blood loss of > 1000 ml (*n* = 146) or ≤ 1000 ml (*n* = 100). Lasso regression analysis was performed on the risk factors screened by univariate analysis to screen out the final risk factors affecting postpartum hemorrhage. Based on the final risk factors, a Nomogram prediction model with excellent performance was constructed using Logistic regression. A nomogram was constructed with further screening of the selected risk factors of postpartum hemorrhage in PPP. A second nomogram based only on the total ultrasonic risk score was constructed. Decision curve analysis (DCA) was used to evaluate the clinical efficacy of the nomograms.

**Results:** Older age, larger gestational age, larger neonatal birth weight, presence of gestational diabetes mellitus, larger amniotic fluid index, absence of gestational bleeding, and higher ultrasonic risk single score were selected to establish a nomogram for postpartum hemorrhage in PPP. The area under the curve of the nomogram constructed by Lasso regression analysis was higher than that of the ultrasonic total score alone (0.887 vs. 0.833). Additionally, DCA indicated better clinical efficacy in the former nomogram than in the later nomogram. Furthermore, internal verification of the nomogram constructed by Lasso regression analysis showed good agreement between predicted and actual values.

**Conclusion:** A nomogram for postpartum hemorrhage in PPP was developed and validated to assist clinicians in evaluating postpartum hemorrhage. This nomogram was more accurate than using the ultrasonic score alone.

## Introduction

Placenta previa is a pregnancy complication in which the placenta (the organ that grows in the uterus to provide oxygen and nutrients to the baby) attaches low within the uterus, covering all or part of the cervix. It is a serious complication of pregnancy; one of the major complications is massive bleeding is massive bleeding during childbirth or the postpartum period, potentially resulting in hysterectomy, blood transfusion, and premature delivery (Oppenheimer, 2007). In 1993, Chattopadhyay et al. (1993) first proposed the concept of pernicious placenta previa (PPP), which refers to placenta previa associated with uterine scar tissue in a woman with a history of cesarean section or myomectomy, and is often accompanied by placenta accreta spectrum disorders (PAS). Clinically, because of a high incidence of PAS, PPP can easily cause fatal and refractory massive bleeding, and even threaten the life of the pregnant woman in serious cases (Zheng et al., 2021). Furthermore, excessive blood transfusion due to bleeding increases the risk of infection and transfusion reaction (Shafer et al., 1980). Additionally, the loss of reproductive opportunities after a hysterectomy in women of childbearing age has great negative impact on their physical and mental health and family life (Horng et al., 2021).

In China, because of “family planning” policies, the cesarean section rate rose rapidly in the 1980s. According to a global survey on maternal perinatal health conducted by the World Health Orga e incidence of caesarean section due to social factors was estimated as approximately 0.01%–2.10% globally and 11.6% in China (Souza et al., 2010). In 2018, Zhang reported that the cesarean section rate in most urban hospitals in China was as high as 40%, and was even 80% in some hospitals (WeiyuanZhang, 2018). Furthermore, due to a recent reform (within the last 5 years) in China’s family planning policies, the number of parturient women has increased, including those with uterine scars. Accordingly, there has been an increase in the incidence of cesarean section scar pregnancy, PAS, uterine rupture, placenta previa, and PPP. Chinese scholars have reported that the current incidence of PPP in China is 0.31%–0.89%, and approximately 53.3% of patients with PPP have accompanying PAS (Yunshan Chen, 2020).

PPP combined with severe intrapartum and postpartum bleeding threatens maternal and infant health, and is one of the great challenges faced by obstetricians in clinical work. In an effort to improve pregnancy outcomes, obstetricians are constantly seeking diagnostic methods that can predict the severity of PPP in order to appropriately intervene and prepare. At present, there are many reports on the prenatal diagnosis of PPP; however, methods to accurately judge and evaluate its severity are lacking. Ultrasound can diagnose about 80% of patients with PPP and is considered by obstetricians to be an ideal method for the prenatal diagnosis of PPP (Jauniaux et al., 2019). Because of its convenient operation, small impact on maternal and fetal health, and capacity for repeatable examinations, without special conditions, it is accepted by most pregnant women. By observing ultrasonic imaging characteristics of the placenta, including relationships between the placenta and cervical opening and, especially, between the placenta and cesarean section scar in the lower section of uterus, and selecting meaningful image features, it is possible to evaluate the type of PPP and whether it is accompanied by PAS (Berkley and Abuhamad, 2018). In addition, ultrasonic images for the diagnosis of placental implantation have the following characteristics: disappearance of the posterior placental space (Pasto et al., 1983), unclear uterine-placenta boundary (Tovbin et al., 2016), interruption or loss of a strong echo line at the uterine serosal laminum-bladder interface (Jauniaux et al., 2018), vortex protrusion (Comstock et al., 2004) and placental echo into the bladder (Wong et al., 2008; Calì et al., 2013), abundant blood flow at the base of the placenta (Finberg and Williams, 1992), and vascular bridge (Finberg and Williams, 1992).

Chinese scholars have proposed a quantitative table based on ultrasound imaging characteristics of the placenta and risk factors of PPP, assigning various scores (Chen et al., 2021). At present, this table is commonly used clinically, and the total score has been widely reported to predict the risk of placenta previa, severe postpartum hemorrhage, hysterectomy, and premature birth in patients with PPP (Chong et al., 2018; Liu et al., 2019; Jingyi Huang and Gu, 2020). However, obstetricians are beginning to pay attention to nomograms for perinatal prediction. The nomograph is a visualization of a complex statistical formula that is increasingly being used in medicine. Nomographs graphically describe a statistical prognostic model that generates the probability of a clinical event (such as cancer recurrence or death) in a particular individual using biological and clinical variables (such as tumor grade and patient age). In order to improve the accuracy of the preoperative assessment of PPP, this study combined reported clinical risk factors with ultrasound scores to construct a model to predict the risk of postpartum hemorrhage in PPP. We consider it more intuitive and concise to predict postpartum hemorrhage in PPP with the developed line diagram, which is convenient for clinical use, than with a quantitative table.

## Materials and methods

### Ethical approval

Ethical approval was obtained from the Ethics Committee of Shengjing Hospital of China Medical University (No. 2022PS132K), and the study conformed to the principles outlined in the Declaration of Helsinki (World Medical Association Declaration of Helsinki).

### Study design

**FIGURE 1:**
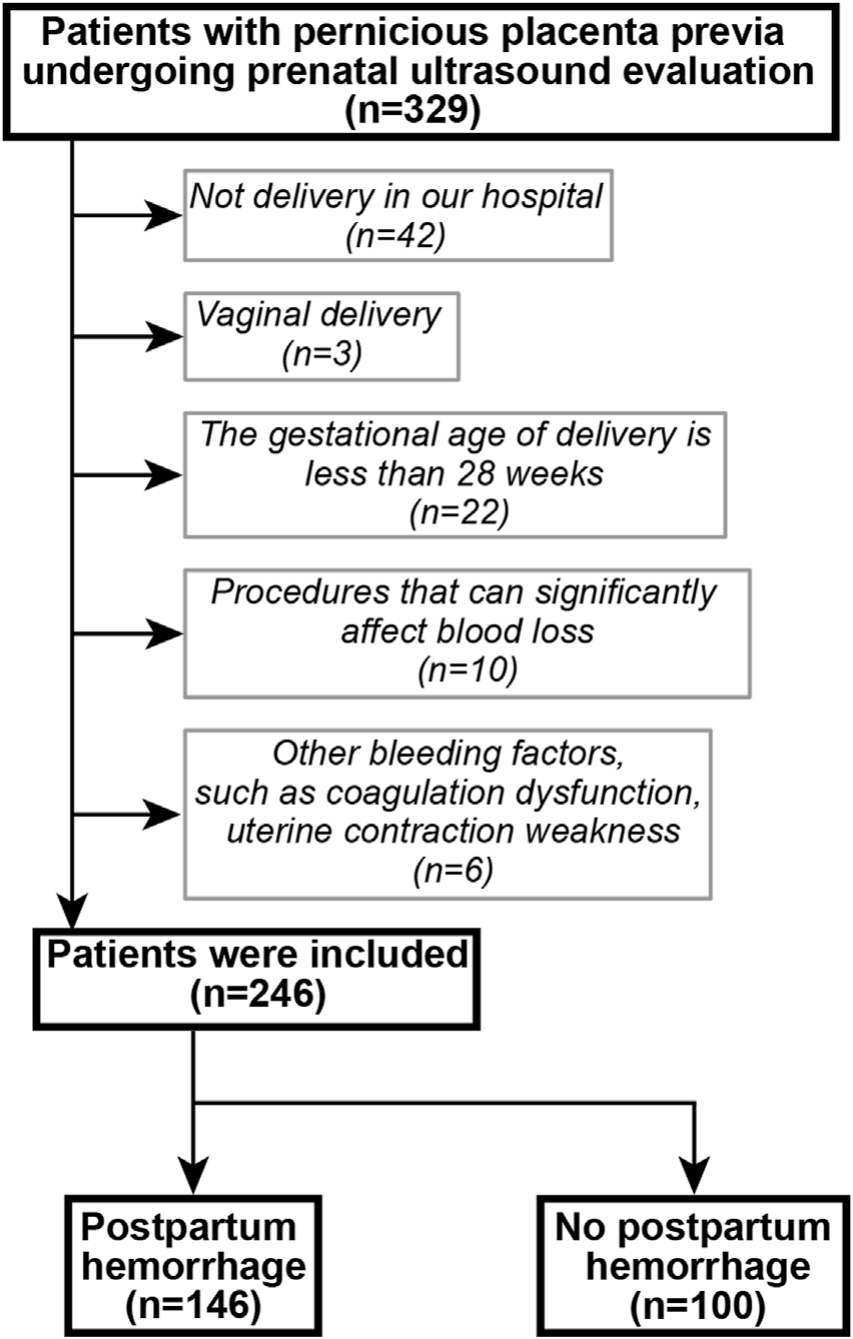
Patient selection flowchart.

A retrospective cohort was established. Patients with suspected PPP who underwent ultrasonography and delivered by cesarean section between January 2018 and December 2019 at the Shengjing Hospital of China Medical University were included. Inclusion criteria were as follows: a history of cesarean section; singleton pregnancy; postoperatively confirmed PPP, with or without PAS; complete clinical data; and ultrasound scoring completed before delivery. The following exclusion criteria were applied: delivery at another hospital; vaginal delivery; gestational age at delivery was less than 28 weeks; underwent procedures that could significantly affect blood loss, such as balloon tamponade, brace sutures, surgical devascularization, radiological embolization and total hysterectomy; presence of a gynecological disease, uterine malformation, uterine fibroids, or adenomyosis; and presence of a disease affecting coagulation function. The patient selection flowchart is shown in Figure 1. PPP with or without PAS disorders were diagnosed using the intraoperative findings or postoperative pathology.

Patient information was collected through the Health Insurance System of the Shengjing Hospital of China Medical University, and included general information (age, ethnicity, times of previous cesarean sections\pregnancies\childbirth\induced abortions, and history of uterine cavity surgery); previous pregnancy information (history of placenta previa, previous operation times); prenatal examination results (placenta previa status and ultrasound examination results in the third trimester); clinical manifestations during pregnancy (prenatal vaginal bleeding); and amount of blood loss during cesarean section. Postpartum hemorrhage was defined as blood loss greater than 1000 ml during cesarean delivery.

### Doppler ultrasound examination

Ultrasound devices (Voluson E10; GE Medical Systems, Milan, Italy) with curved probes (1–5 MHz) and endovaginal transducers (5–9 MHz) were used. Before the examination, the patient, with a full bladder, was placed in a supine position. Patients were scanned for placental location and thickness, placental echo, placental margin, and uteral margin and thickness. When the placenta was located at the base of the anterior wall, the relationship between the continuity of the posterior wall and bladder wall was specifically scanned. Sonographic scores were computed using complete scan data, including location of the placenta, placental thickness, continuity of the clear space, bladder line, placental lacunae, condition of the subplacental vascularity, and cervical morphology, cervical sinus blood, as well as the number of pervious cesarean deliveries (Chen et al., 2021) (Table 1).

**TABLE 1:** Sonographic scoring system for pernicious placenta previa.

Item	0	1	2
Location of the placenta	Normal	Marginal placental previa or low lying placenta	Complete placental previa
Placental thickness (cm)	<3	3–5	>5
Loss of clear zone	Continuity	Local interruption	Disappeared
Bladder line	Continuity	Local interruption	Disappeared
Placental Lacunae	None	Present	Fusion, with “boiling water sign”
Condition of the subplacental vascularity	Normal blood flow	Increased, forming a cluster	“Transboundary” blood vessels
Cervical blood sinus	None	Present	Fusion, with “boiling water sign”
Cervical morphology	Complete	Incomplete	Disappeared
Pervious cesarean deliveries (number)	None	1	≥2

### Statistical analysis

Analyses were performed in the R-Studio environment using R (version 3.6.3; R Foundation for Statistical Computing, Vienna, Austria; http://www.r-project.org). In order to identify the risk factors of postpartum hemorrhage, the odds ratio (OR) and 95% confidence interval (CI) were calculated for each clinical factor using univariate logistics regression analysis. Lasso regression analysis was performed on the risk factors screened by univariate analysis to screen out the final risk factors affecting postpartum hemorrhage. Logical Lasso regression modeling is a shrinkage approach that actively selects from a large and potentially multicollinear set of variables to produce a more relevant and interpretable set of predictive variables (Muthukrishnan and Rohini, 2016). We used tenfold cross validation to select the penalty term, *λ*. The built-in function in R produces two automatic λs, one of which minimizes the binomial bias, rendering the covariables included in the study more comprehensive. We also constructed a nomogram of postpartum hemorrhage based entirely on the total ultrasound risk score The prediction ability of the nomograms was evaluated by the area under the receiver operating characteristic (ROC) curve (AUC); AUCs closer to 1.0 are considered to have better recognition ability (Obuchowski and Bullen, 2018). The nomograms were internally verified by bootstrapping (1,000 resamplings) (Henderson, 2005). Decision curve analysis (DCA) (Vickers et al., 2019) was used to calculate the total benefit at each possible risk threshold and evaluate the clinical efficacy of the nomogram. Statistical significance was set as *p* < 0.05.

## Results

### Patient characteristics

During the study period, 329 patients underwent ultrasound examination for suspected PPP. After applying the exclusion criteria, 246 patients remained and were included in the study. Of these, 146 patients experienced postpartum bleeding. Specific patient characteristics are shown in Table 2.

**TABLE 2:** Characteristics of patients with pernicious placenta previa.

Characteristic	No postpartum hemorrhage	Postpartum hemorrhage	*p-*value
	N = 100	N = 146	
Age (years)			0.013
<30	28 (28%)	23 (16%)	
30–35	48 (48%)	80 (55%)	
36–39	18 (18%)	30 (21%)	
≥40	6 (6.0%)	13 (8.9%)	
Gestational age (weeks)			0.019
<37	52 (52%)	55 (38%)	
≥37	48 (48%)	91 (62%)	
Number of pregnancies			0.973
2	30 (30%)	48 (33%)	
3	25 (25%)	38 (26%)	
4	21 (21%)	24 (16%)	
≥5	24 (24%)	36 (25%)	
Number of abortions			0.618
None	30 (30%)	55 (38%)	
1–2	50 (50%)	68 (47%)	
≥3	20 (20%)	23 (16%)	
Neonatal weight (g)			<0.001
<2500	47 (47%)	36 (25%)	
2500–4000	52 (52%)	103 (71%)	
>4000	1 (1.0%)	7 (4.8%)	
PIH			0.965
No	98 (98%)	142 (97%)	
Yes	2 (2.0%)	4 (2.7%)	
GDM			0.004
No	93 (93%)	119 (82%)	
Yes	7 (7.0%)	27 (18%)	
Amniotic fluid index			0.042
<5	10 (10%)	5 (3.4%)	
5–18	89 (89%)	136 (93%)	
>18	1 (1.0%)	5 (3.4%)	
Time from previous cesarean section (years)			0.967
1–5	56 (56%)	79 (54%)	
6–10	34 (34%)	55 (38%)	
>10	10 (10%)	12 (8.2%)	
Vaginal bleeding during pregnancy			<0.001
None	49 (49%)	106 (73%)	
Yes	51 (51%)	40 (27%)	
Fetal position			0.700
Cephalic presentation	78 (78%)	122 (84%)	
Breech presentation	13 (13%)	16 (11%)	
Transverse lie presentation	9 (9.0%)	8 (5.5%)	
Ultrasound risk score—position of the placenta			<0.001
1	12 (12%)	3 (2.1%)	
2	88 (88%)	143 (98%)	
Ultrasound risk score—thickness of the placenta			<0.001
0	16 (16%)	12 (8.2%)	
1	78 (78%)	86 (59%)	
2	6 (6.0%)	48 (33%)	
Ultrasound risk score—continuity of the clear space			<0.001
0	34 (34%)	17 (12%)	
1	27 (27%)	39 (27%)	
2	39 (39%)	90 (62%)	
Ultrasound risk score—bladder line			<0.001
0	56 (56%)	27 (18%)	
1	25 (25%)	43 (29%)	
2	19 (19%)	76 (52%)	
Ultrasound risk score—lacunae			<0.001
0	73 (73%)	55 (38%)	
1	22 (22%)	55 (38%)	
2	5 (5.0%)	36 (25%)	
Ultrasound risk score—condition of the subplacental vascularity			<0.001
0	24 (24%)	6 (4.1%)	
1	33 (33%)	42 (29%)	
2	43 (43%)	98 (67%)	
Ultrasound risk score—cervical blood sinus			<0.001
0	92 (92%)	104 (71%)	
1	6 (6.0%)	18 (12%)	
2	2 (2.0%)	24 (16%)	
Ultrasound risk score—cervical morphology			<0.001
0	93 (93%)	109 (75%)	
1	5 (5.0%)	22 (15%)	
2	2 (2.0%)	15 (10%)	
Ultrasound risk score—pervious cesarean deliveries (number)			<0.001
1	94 (94%)	116 (79%)	
2	6 (6.0%)	30 (21%)	

PIH, pregnancy-induced hypertension syndrome; GDM, gestational diabetes mellitus.

### Selected risk factors

**FIGURE 2:**
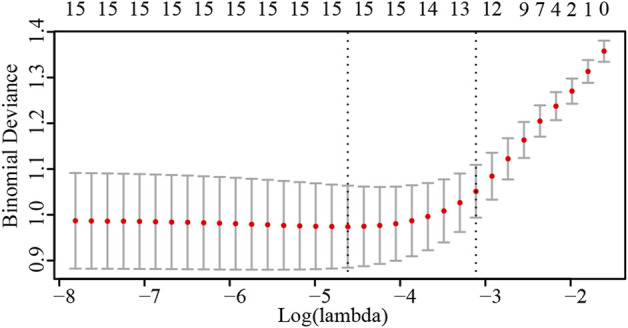
Number of risk factors determined by cross-validation of penalty terms in Lasso regression analysis.

Results of the univariate logistic regression and Lasso regression analyses of postpartum hemorrhage in PPP are shown in Table 3; Figure 2. In the final Lasso analysis, older age, larger gestational age, larger neonatal birth weight, presence of gestational diabetes mellitus (GDM), larger amniotic fluid index, absence of gestational bleeding, and higher ultrasound risk single score were associated with a higher risk of postpartum hemorrhage.

**TABLE 3:** Univariate logistic and Lasso regression analyses of postpartum hemorrhage in pernicious placenta previa.

Characteristic	Univariate logistic regression			
	OR	95% CI	*p*-value	Lasso coefficient
Age	1.36	1.02, 1.88	0.048*	0.140
Gestational age	1.79	1.07, 3.01	0.027*	0.112
Number of pregnancies	0.96	0.77, 1.19	0.685	—
Number of abortions	0.78	0.54, 1.13	0.191	—
Neonatal weight	2.66	1.61, 4.47	<0.001*	0.699
PIH	1.38	0.26, 10.1	0.713	—
GDM	3.01	1.32, 7.79	0.013*	0.665
Amniotic fluid index	3.10	1.23, 8.89	0.022*	0.800
Time from previous cesarean section	1.00	0.68, 1.49	0.990	—
Vaginal bleeding during pregnancy	0.36	0.21, 0.62	<0.001*	−0.516
Fetal position	0.76	0.49, 1.18	0.225	—
Ultrasound risk score—position of the placenta	6.50	2.00, 29.1	0.005*	1.470
Ultrasound risk score—thickness of the placenta	3.31	1.99, 5.78	<0.001*	0.754
Ultrasound risk score—continuity of the clear space	2.07	1.49, 2.90	<0.001*	0.258
Ultrasound risk score—bladder line	2.90	2.08, 4.13	<0.001*	0.289
Ultrasound risk score—lacuna	3.18	2.11, 4.96	<0.001*	0.633
Ultrasound risk score—condition of the subplacental vascularity	2.52	1.72, 3.76	<0.001*	0.463
Ultrasound risk score—cervical blood sinus	3.04	1.79, 5.86	<0.001*	0.740
Ultrasound risk score—cervical morphology	2.96	1.64, 6.11	0.001*	0.382
Ultrasound risk score—ceasarean delivery (number)	4.05	1.73, 11.2	0.003*	1.102

PIH, pregnancy-induced hypertension syndrome; GDM, gestational diabetes mellitus; OR, odds ratio; CI, confidence interval; Ref, reference; *, *p* < 0.05.

### Nomogram construction

**FIGURE 3:**
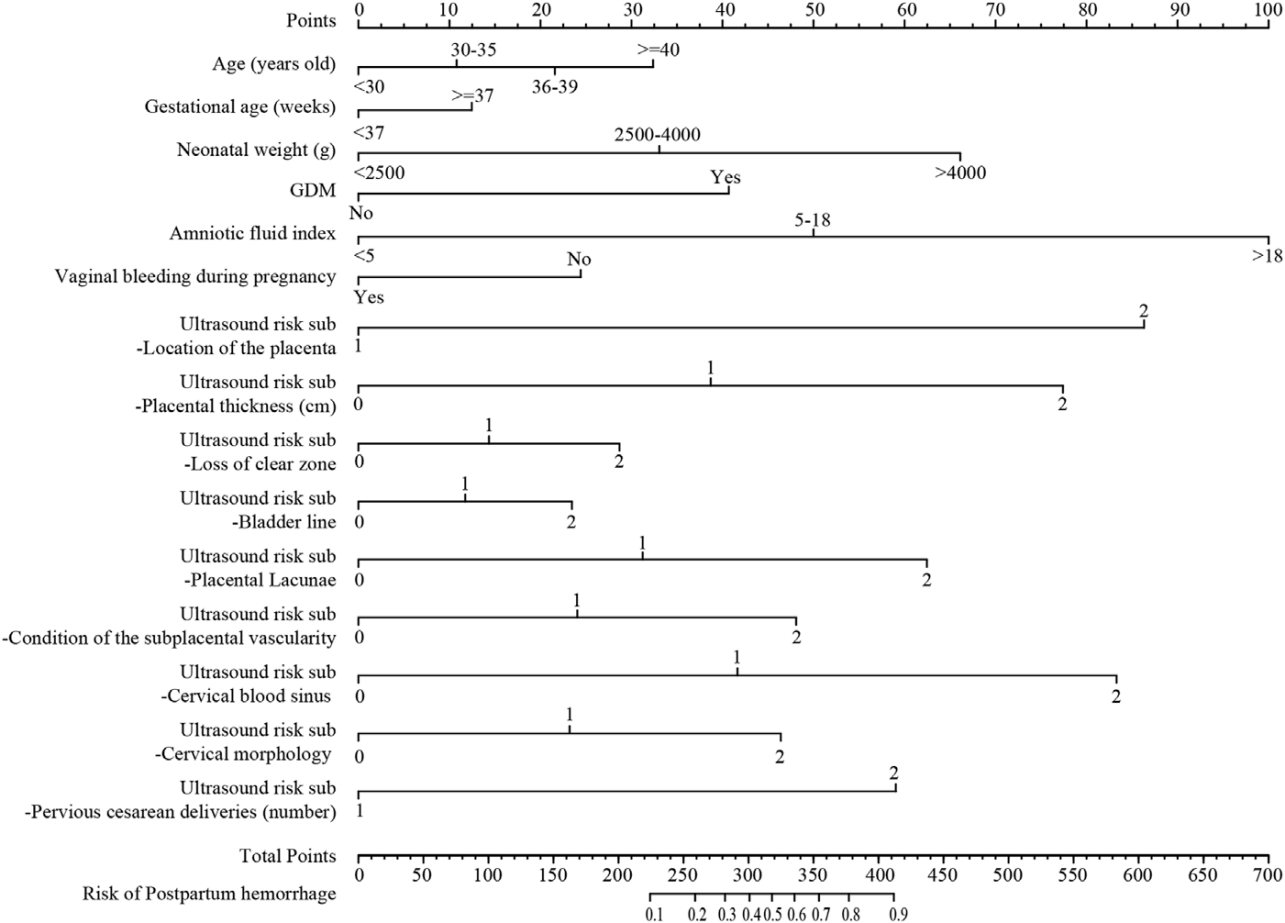
The nomogram of postpartum hemorrhage based on the Lasso regression analysis. GDM, gestational diabetes mellitus.

The nomogram of postpartum hemorrhage based on variables from the Lasso regression analysis, including age, gestational age, neonatal birth weight, GDM, amniotic fluid index, gestational bleeding, and ultrasound risk single score is shown in Figure 3. The nomogram of postpartum hemorrhage based entirely on the ultrasonic risk total score is shown in Supplementary Material S1.

### Nomogram performance

**FIGURE 4:**
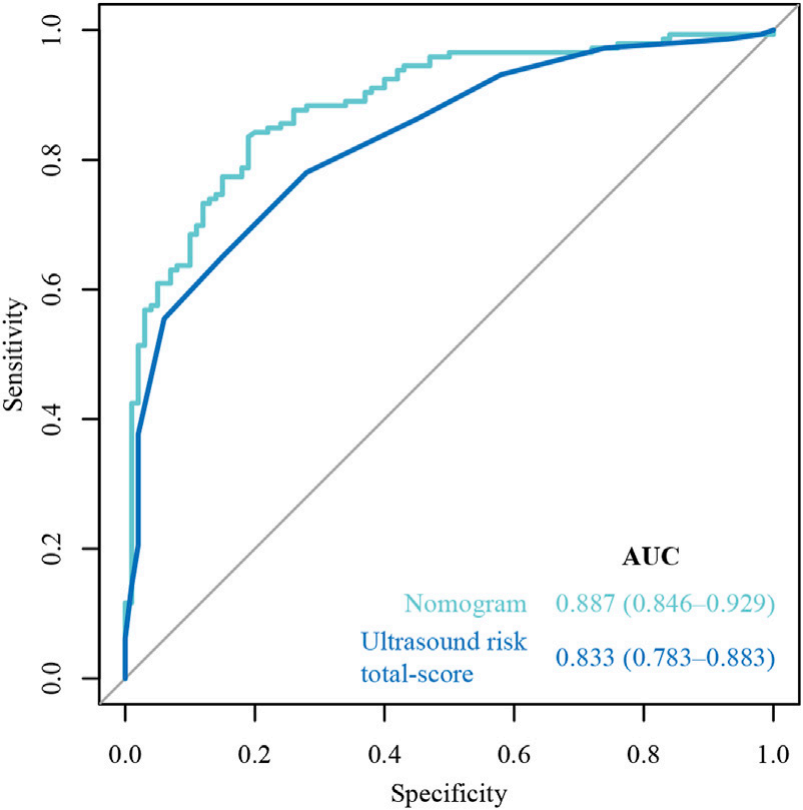
Receiver operating characteristic (ROC) curve of the nomograms.

**FIGURE 5:**
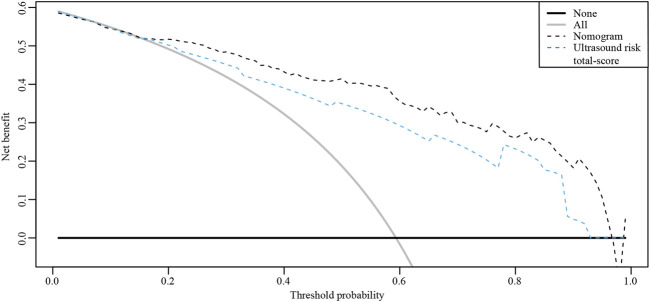
Decision curve analysis (DCA) of the nomograms.

**FIGURE 6:**
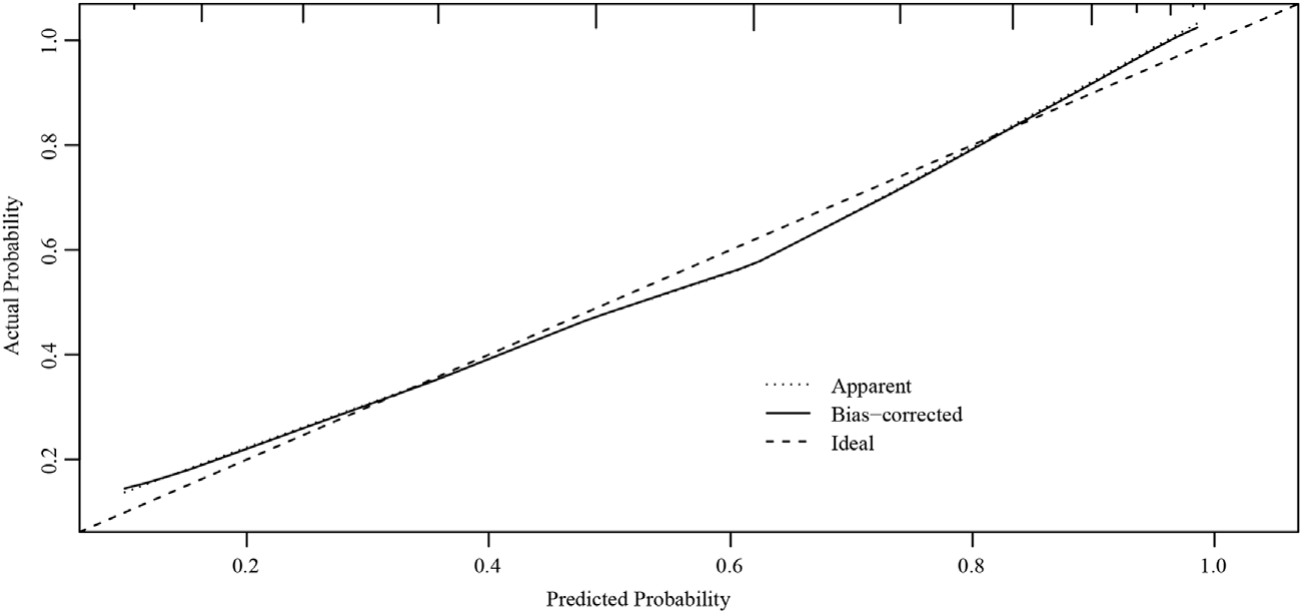
Internal verification plots of the nomogram calibration curves by bootstrapping with 1000 resamples.

The ROC curves of the nomograms of postpartum hemorrhage are shown in Figure 4. The AUC of the nomogram constructed by Lasso regression analysis was higher than that of the nomogram constructed by the ultrasonic risk total score alone, suggesting that the former nomogram has a better ability to predict postpartum hemorrhage than the latter nomogram. In addition, DCA indicated that the former nomogram has better clinical efficacy than the latter nomogram (Figure 5). Internal validation of the nomogram based on Lasso regression analysis showed a calibration curve close to 45 degrees (Figure 6), indicating good agreement between predicted and actual values.

## Discussion

In 2016, the Chinese scholar, Yang, and colleagues reported that the average incidence of PPP at Peking University Third Hospital was 2.08/1000, which increased each year from 0.9/1000 in 2008 to 3.08/1000 in 2014. In addition, 53.3% of patients had placenta implantation, and 90% of patients suffered bleeding of more than 3,000 ml during surgery (Lin Yu et al., 2016). At present, research on a prenatal diagnostic method for PPP is an important and active field in obstetrics worldwide. However, due to a lack of objective indicators for an accurate preoperative judgment and evaluation of the degree of risk in PPP, preoperative preparation is often inadequate or excessive, resulting in unnecessary hysterectomy or a waste of medical resources.

The main cause of PPP postpartum hemorrhage is placental implantation, which is collectively referred to in the 2018 FIGO guidelines as placenta accreta spectrum (PAS) (Hecht et al., 2020). Numerous studies on the ultrasonic diagnosis of PAS, have reported diagnostic sensitivities ranging from 50% to 87% (Jauniaux et al., 2018; D’Antonio et al., 2013; Jauniaux and Bhide, 2017). Therefore, regardless of the imaging method, the prenatal diagnosis of PPP with/without PAS is relatively subjective, and its accuracy depends largely on the experience of the operator. A study published by Dimitrova and colleagues suggested that the diagnostic accuracy of PPP was higher in ultrasound operators who received standard training than in ultrasound operators who only received basic obstetrics training (Dimitrova et al., 2019). Naturally, in addition to subjective factors, some objective factors, such as subcutaneous fat and poor patient compatibility, contribute to the occurrence of missed diagnoses and the misdiagnosis of ultrasound results. A meta-analysis conducted by Xu and colleagues showed that magnetic resonance imaging was more effective than ultrasound in the prenatal diagnosis of PPP (Zeng et al., 2018).

Zhao and colleagues published an ultrasonic scoring system for PAS in 2018, which can not only predict the type of PAS, but also predict the risk of intraoperative bleeding and hysterectomy (Chong et al., 2018). Higher PAS scores indicate higher risk of intraoperative hemorrhage and hysterectomy. By drawing an ROC curve, the cut-off values of PAS were determined by a score of 5 for placenta increta (PI) and 10 for placenta percreta (PP). The scale is easy to operate and understand by using typical signs of placenta implantation as the prediction standard, combined with the high-risk factor of a previous cesarean section, to evaluate postpartum hemorrhage in PPP with specific scores. Use of this scale can effectively avoid the influence of subjective factors and has been promoted and recognized in China’s clinical practice (Lingyan Zhu et al., 2018; Hui Cen et al., 2019; Zhu and Xie, 2019). Building upon this scale, the present study combined ultrasonic data with clinical characteristics; all factors were fitted and quantified to establish a prediction model. Its accuracy was significantly higher than that of the ultrasonic risk total score, and it had better application value.

Calì and colleagues (Calì et al., 2013) reported that hypervascularity of the entire uterine serosa-bladder wall interface showed a sensitivity, specificity, negative predictive value, and positive predictive value of 90%, 100%, 100%, and 97%, respectively, in 17 PP cases. Furthermore, irregular intraplacental vascular formation and curved vessels affecting the entire width of the placenta showed high specificity (100%) and positive predictive value (100%). In a retrospective analysis of ultrasound imaging data from 232 pregnant women with high-risk factors for PAS, Chalubinski et al. (2013) predicted the implant type based on specific recognized ultrasound signs for PAS diagnosis, with clinical outcomes and pathological returns as the final evaluation criteria; this method did not misdiagnose PI or PP as normal placenta or PA, and the accuracy of screening reached 100%. Han *et al* (Penghui Han et al., 2016) reported that the degree of abnormal thickening of blood vessels in the placenta and the size of the blood sinus may be related to the range and depth of PA. Furthermore, the report by Yanmei Liu et al. (2017) suggested that the risk of hysterectomy during cesarean section was very high when the ultrasound examination found extensive lacunae in the placenta, focal or extensive lacunae in the placenta with visible blood flow, and abundant blood flow at the serum-bladder junction.

In addition, many researchers have reported on the risk factors of PPP accompanied by PAS, but controversy still exists. The reported risk factors mainly comprised old age, history of multiple uterine surgeries, history of multiple abortions, intrauterine membrane inflammation, and maternal lifestyle habits such as smoking (Eshkoli et al., 2013). Furthermore, medicines used during pregnancy may lead to PA by affecting the secretion of some factors in the placenta, thus precipitating trophoblast cell invasion and abnormal angiogenesis of the placenta. In addition, medicines such as heparin and aspirin may affect blood circulation and cytokine secretion throughout the body, which may lead to changes in maternal endometrial hormone levels, which in turn affect endometrial decidualization, leading to PAS (Niringiyumukiza et al., 2018). Additionally, the study by Bowman et al. (2014) showed that the number of previous cesarean sections was a risk factor affecting the degree of PPP. In the present study, postpartum hemorrhage in PPP was associated with neonatal weight, GDM, and vaginal bleeding during pregnancy.

At present, there are few reports on the relationship between vaginal bleeding in early pregnancy and PAS/PPP. Multivariate logistic regression analysis in Chou et al. (2000) identified a history of vaginal bleeding in early pregnancy as an independent risk factor for PAS (*p* < 0.05), which significantly increased the risk of placental implantation approximately fivefold (OR = 5.336, 95% CI 1.874–15.197). Wu and Yan, 2015 reported that slight vaginal bleeding in early pregnancy was related to embryo implantation, but long-term and severe bleeding could cause pathological damage to the decidua and placenta, thus reducing the physiological function of the placenta and ultimately leading to pregnancy complications such as PAS. There are also studies that patients with PI and PP, but not easy to cause vaginal bleeding (Bhide et al., 2019). In the present study, vaginal bleeding during pregnancy was an independent risk factor for postpartum hemorrhage in PPP, and patients with PPP with vaginal bleeding during pregnancy had a lower rate of postpartum bleeding, which is consistent with the results of Bhide et al. (2019).

Additionally, GDM is an independent risk factor for postpartum hemorrhage in PPP. There are numerous studies on angiogenesis and vascular remodeling associated with the placenta in GDM. The most common placental changes associated with GDM are chorangiosis (Stanek, 2016) and placental venous immaturity (Ogino and Redline, 2000; Rossi et al., 2012; Soma et al., 2013), and GDM is associated with accelerated microangiopathy. The number of villous capillaries is increased, especially in the center of the villi, in the placenta in GDM. Daskalakis et al. (2008) investigated 40 placentae associated with GDM and found that 40% of placentae showed chorangiosis. The proliferation of capillaries and pathophysiological changes in the placenta may increase the risk of postpartum hemorrhage in PPP.

Due to the establishment of inclusion and exclusion criteria and the limited number of PPP patients, the study results have some limitations. The ultrasound-based nomogram was designed to predict the risk of postpartum bleeding and the risk of adverse outcomes in PPP, should be verified in prospective cohort in the future. In addition, the prediction efficiency of different ultrasonic signs for PPP combined with PAS is different. If multicenter studies are included in which different intraoperative treatments are used, the current predictions might have changed considerably.

## Conclusion

Our ultrasound-based nomogram can be used to predict postpartum hemorrhage and the risk of adverse outcomes in PPP. Our nomogram can aid clinicians in accurately assessing the severity of PPP before surgery, to determine the best time to terminate pregnancy according to clinical characteristics and ultrasound results, and fully prepare their personnel and supplies before surgery. The planned organization of a multiple disciplinary team with member from obstetrics, neonatology, anesthesiology, the intensive care unit, interventional radiology, and the blood bank can improve the outcomes of PPP as much as possible, while ensuring the safety of patients, taking into account fetal maturity and post-birth survival.

## Data Availability

The raw data supporting the conclusion of this article will be made available by the authors, without undue reservation.

## Abbreviations

AUC, area under the receiver operating characteristic curve; CI, confidence interval; DCA, decision curve analysis; GDM, gestational diabetes mellitus; OR, odds ratio; PA, placenta accrete; PAS, placenta accreta spectrum; PI, placenta increta; PP, placenta percreta; PPP, pernicious placenta previa; ROC, receiver operating characteristic.
