# Supplementary figures and images for: Ultrasound-based nomogram for postpartum hemorrhage prediction in pernicious placenta previa
Source: Front Physiol. 2022 Aug 22;13:982080. doi: 10.3389/fphys.2022.982080 (PMC9441797; doi:10.3389/fphys.2022.982080)

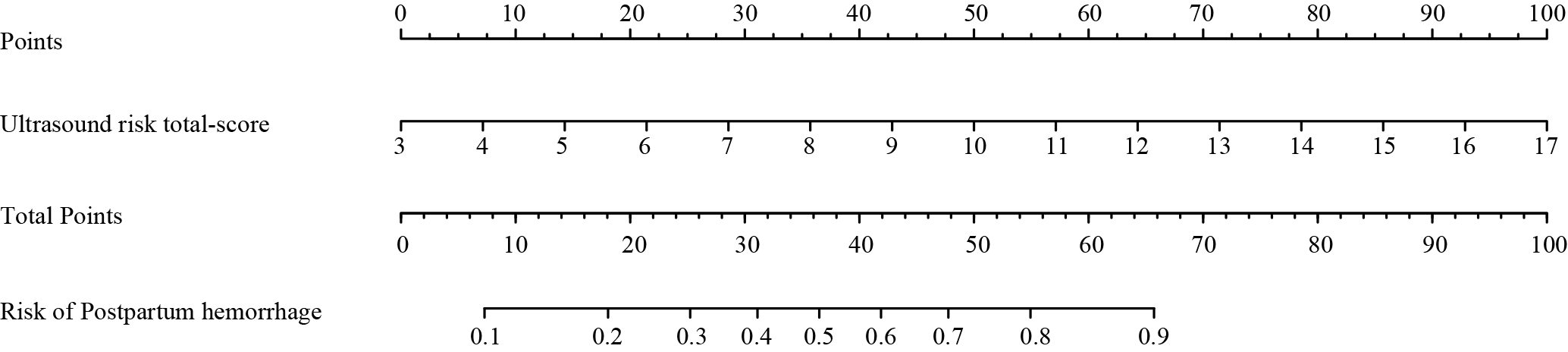

Supplement: Supplementary file 1 [file Image1.TIF]
